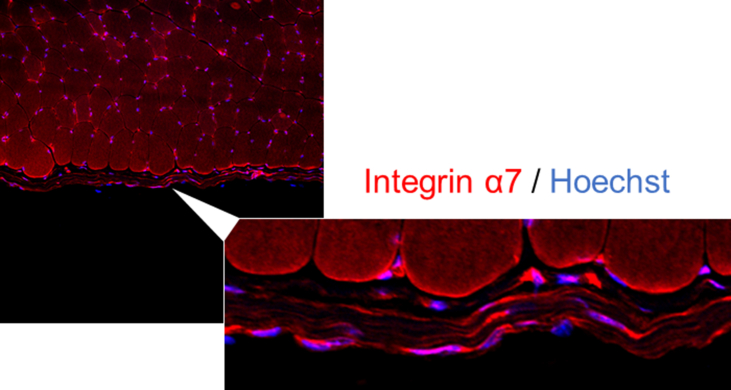# Laminin-221-derived recombinant fragment facilitates isolation of cultured skeletal myoblasts

**DOI:** 10.1016/j.reth.2022.04.006

**Published:** 2022-05-12

**Authors:** Yuki Kihara, Jun Homma, Ryo Takagi, Keiko Ishigaki, Satoru Nagata, Masayuki Yamato

**Affiliations:** a Department of Pediatrics, Tokyo Women's Medical University, School of Medicine, 8-1 Kawada-cho, Shinjuku-ku, Tokyo, 162-8666, Japan; b Institute of Advanced Biomedical Engineering and Science, Tokyo Women's Medical University, 8-1 Kawada-cho, Shinjuku-ku, Tokyo, 162-8666, Japan

**Keywords:** Primary culture, Myoblast, Laminin-221, Integrinα7X2, Skeletal muscle, ECM, extracellular matrix, FACS, fluorescence-activated cell sorting, MACS, magnetic-activated cell sorting, PBS, Dulbecco's phosphate buffer saline, GM, growth medium, DM, differentiation medium, PFA, paraformaldehyde

## Abstract

**Introduction:**

Laminin is a major component of the basement membrane, containing multiple domains that bind integrin, collagen, nidogen, dystroglycan, and heparan sulfate. Laminin-221, expressed in skeletal and cardiac muscles, has strong affinity for the cell-surface receptor, integrin α7X2β1. The E8 domain of laminin-221, which is essential for cell integrin binding, is commercially available as a purified recombinant protein fragment. In this study, recombinant E8 fragment was used to purify primary rodent myoblasts. We established a facile and inexpensive method for primary myoblast culture exploiting the high affinity binding of integrin α7X2β1 to laminin-221.

**Methods:**

Total cell populations from dissociated muscle tissue were enzymatically digested and seeded onto laminin-221 E8 fragment-coated dishes. The culture medium containing non-adherent floating cells was removed after 2-hour culture at 37 °C. The adherent cells were subjected to immunofluorescence staining of desmin, differentiation experiments, and gene expression analysis.

**Results:**

The cells obtained were 70.3 ± 5.49% (n = 5) desmin positive in mouse and 67.7 ± 1.65% (n = 3) in rat. Immunofluorescent staining and gene expression analyses of cultured cells showed phenotypic traits of myoblasts.

**Conclusion:**

This study reports a novel facile method for primary culture of myoblasts obtained from mouse and rat skeletal muscle by exploiting the high affinity of integrin α7X2β1 to laminin-221.

## Highlights

• Myoblasts are muscle progenitor cells that differentiate into skeletal muscle.
• Various methods have been reported to isolate myoblasts, such as FACS and MACS.
• Integrin α7X2, predominantly expressed in myocytes and cardiomyocytes, binds laminin-221 with high affinity.
• We established a novel method for primary culture of myoblasts by utilizing the high affinity of integrin α7X2β1 to laminin-221.

## 1 Introduction

The basement membrane is a specialized form of the extracellular matrix (ECM) that surrounds muscle fibers. Cell adhesion to the basement membrane is involved in the proliferation and apoptosis of cells [1]. Major components of the basement membrane are type IV collagen, proteoglycans, and glycoproteins such as laminin. The laminin protein family is a diverse set of 11 proteins commonly having three polypeptide chains (α, β, and γ) connected with disulfide bonds [2]. Integrins and dystroglycans are the corresponding cell-surface receptors. Integrin-specific binding of laminin serves as a transmembrane linker which connects the cytoskeleton to the ECM, producing mechanical functions as well as prompting outside-in cell signaling. Thus, integrins and laminins control cell behavior including cell migration, differentiation, and proliferation through ECM-binding [1]. Integrin α7β1 is predominantly expressed in skeletal and cardiac muscles that originate from mesoderm. Two different sequences (X1 and X2) are located near the ligand binding site of the integrin α7 subunit. These domains are derived from the same gene by mutually exclusive alternative mRNA splicing and are equally expressed in myoblasts and the myocardium [3]. Integrin α7X1β1 binds to all laminins except laminin-332, which is found in cutaneous epithelial basement membrane; however, it preferentially binds laminin-211/221 and laminin-511/521 in muscle basement membrane, while α7X2β1 preferentially binds laminin-111 and laminin-211/221 [4].

We hypothesized that by exploiting the higher binding capacity of integrin α7X2β1 expressed by myoblasts to laminins, they could be selectively isolated from skeletal muscle tissues and their successful primary culture may be established on laminin substrates. Previous studies have reported myoblast cultures on laminin-111 [5] and Matrigel® [6]. However, laminins are large complex glycoproteins (900 kDa), which are difficult to isolate and purify, and recombinant laminin production is expensive. Laminin-111 isolated from Matrigel® is commercially available as an extract from murine Engelbreth-Holm-Swarm tumor. From the viewpoints of practicality and safety, its use in clinical settings for myoblast regeneration might be limited. Therefore, in the present study, a commercial human laminin-derived recombinant protein fragment was utilized. The fragment was produced by Chinese hamster ovary (CHO) cells transfected with genes encoding the E8 domain of laminin-221 that is essential for integrin binding. Laminin E8 fragments are truncated proteins consisting of the C-terminal regions of α, β, and γ chains. This truncated protein contains active integrin-binding sites such as laminin globular 1–3 domain of the α chain and the glutamate residue in the C-terminal tail of the γ chain. Moreover, the E8 fragment does not bind to heparin and heparan sulfate unlike full-length laminin. Thus, it is the smallest unit with integrin-binding ability [7].

In the current rapidly aging society, age-related muscle atrophy and fragility (sarcopenia) remain of increasing medical concern. Few clinical options are available for treating various sarcopenias. These include nutrition and exercise intervention, but their effects are limited [8]. Therefore, improved understanding of the underlying molecular machinery in muscle pathology and muscle regeneration and the development of better treatments are needed. Muscle regenerative medicine for sarcopenia has not been reported in humans [9]; however, treatment for muscular dystrophies and muscle injuries has already been reported. In a study of immunosuppressed patients with Duchenne muscular dystrophy, when myoblasts were intramuscularly injected, dystrophin expression was observed in a small number of donor-derived myocytes [10]. In a study in which myoblasts were intramuscularly injected in the pharyngeal muscle of patients with oculopharyngeal muscular dystrophy, improvement in the quality of life and volume-dependent recovery of swallowing function were observed [11]. Up to now, the therapeutic effect is still limited, and further studies are needed. Facile primary skeletal myoblast culture methods should be established as a basis for improving skeletal muscle regenerative medicine and developing molecular medicine approaches for muscular diseases.

Currently, primary culture of skeletal myoblasts is performed using several methods for myoblast isolation. Previously reported methods include the explant cell culture method [12] in which myoblasts migrate from biopsied muscle fragments on culture surfaces at early time points due to the high intrinsic myoblast motility. Percoll density gradient-based cell separation [13] is used to eliminate other cell types from myoblasts. Fluorescence-activated cell sorting (FACS) and magnetic-activated cell sorting (MACS) by negative selection using antibodies against well-known cell-surface markers such as Sca-1 CD31, CD45, and CD11b for isolated myoblasts; and positive selection using anti-α7 integrin antibody have also been reported [14,15]. To eliminate fibroblasts, a major contaminant in muscle-derived cell populations, differences in cell adhesion capacity to collagen are also utilized [16]. Notably, MACS and FACS are most commonly used because the other methods are often time-consuming with poor myoblast purity. However, antibodies for cell selection are sometimes species-specific, and their applications to some experimental animal models and human clinical settings are limited. Furthermore, antibodies and the required equipment for this cell sorting are often expensive.

To overcome these shortcomings of conventional methods for myoblast isolation, we utilized laminin-derived recombinant fragment-coated isolation and culture surfaces. By simply plating total cell populations obtained from minced and enzymatically digested skeletal muscle and washing non-adherent floating cells from laminin-coated dishes, cells other than myoblasts were eliminated. Further, isolated myoblasts were subjected to prolonged cell culture and differentiation on these same surfaces. To demonstrate species-independence of this method, both rat and mouse myoblasts were successfully isolated from their skeletal muscles with high cell purity.

## 2 Materials and methods

### 2.1 Ethics

All experiments were approved by the Ethics Committee of Tokyo Women's Medical University, Tokyo, Japan, and animal care was based on guidelines from the Science Council of Japan. This study was carried out in compliance with the Animal Research: Reporting of in Vivo Experiments (ARRIVE) guidelines.

### 2.2 Animals

C57BL/6J mice and Sprague Dawley (SD) rats were purchased from Sankyo Lab Service Corporation, Inc., Japan. Mice were housed in the Institute of Laboratory Animals, Tokyo Women's Medical University. Rats were housed in the Institute of Advanced Biomedical Engineering and Science, Tokyo, Japan. Both rodent species were housed in separate cages, with no more than 5 mice/cage and no more than 2 rats/cage, with 12-hour light/dark cycles.

### 2.3 Preparation of human laminin-221-derived recombinant fragment-coated surfaces

Human laminin-221-derived recombinant fragment (iMatrix-221, Nippi Inc., Tokyo, Japan) was provided in vials as a solution in Dulbecco's phosphate buffer saline (PBS, FUJIFILM Wako Pure Chemical Corporation, Osaka, Japan). Each vial was diluted using 11 mL PBS made to a final concentration of 5 μg/mL. The total volume was added into 100-mm Primaria cell culture dishes (Corning, New York, USA) for isolation and prolonged culture of myoblasts, and incubated at 37 °C for 1.5 h.

For initial primary cell adhesion assays, 2 μL iMatrix-221 solution was diluted with 420 μL PBS to a final concentration of 2.38 μg/mL, and then its 2 μL aliquot was added into wells of 24-well Primaria culture plates (Corning) at 0.5 μg/cm^2^, and incubated at 37 °C for 1.5 h. After discarding the solution, 3 mL 1% BSA (Sigma-Aldrich, St. Louis, USA) in PBS was added into the wells and incubated at 37 °C for 45 min for surface blocking. These procedures were performed immediately before cell culture use; thus, the culture surfaces used were always wet and never desiccated.

### 2.4 Cell culture

Cell culture for all cell isolates was performed using the following culture media: 1) Ham's F-10 Nutrient Mix (Life Technologies, Carlsbad, Canada) supplemented with 2 ng/mL basic fibroblast growth factor (FUJIFILM Wako Pure Chemical Corporation), 10% fetal bovine serum (FBS, Life Technologies), and 1% penicillin-streptomycin (FUJIFILM Wako Pure Chemical Corporation) for myoblast growth medium (GM); 2) GM without FBS for myoblast isolation cultures to eliminate any effects of FBS-contained fibronectin; 3) high-glucose Dulbecco's Modified Eagle Medium (FUJIFILM Wako Pure Chemical Corporation), supplemented with 2% horse serum (Life Technologies) and 1% penicillin-streptomycin differentiation medium (DM) for skeletal muscle differentiation cultures.

### 2.5 Automated computerized cell counting

In the initial cell adhesion and cell proliferation assays, cell numbers on wells were automatically counted. After each denoted culture period, wells were gently washed with PBS to remove non-adherent, floating cells; adherent cells were fixed with 4% paraformaldehyde (PFA) in PBS and washed with only PBS. Then, the cell nuclei were stained with a DNA-binding fluorescent dye (Hoechst 33258, Life Technologies), and the cells were washed. Cell nuclei were automatically counted using Image Xpress Ultra and MetaXpress Image Acquisition software (Molecular Devices, San jose, USA).

### 2.6 Primary culture of skeletal muscle-derived cells by MACS

Experimental animals (six-week-old C57BL/6J mice or four-week-old SD rats) were anesthetized with isoflurane and sacrificed by exsanguination. Connective tissue, blood vessels, and fat were carefully removed from muscle collected from the lower limbs using forceps under a stereomicroscope. The collected muscle tissue samples were placed in Hank's balanced salt solution (HBSS-, FUJIFILM Wako Pure Chemical Corporation) containing 1% penicillin-streptomycin and gently shaken to avoid contamination. Myoblast-containing cell suspensions were prepared using the MACS skeletal muscle dissociation kit for mouse and rat (Miltenyi Biotec, North Rhine, Germany). Control mouse myoblasts were isolated by MACS with a purity of 98.5 ± 0.208% (n = 3), using the satellite cell isolation kit (Miltenyi Biotec, North Rhine, Germany), according to the manufacturers' protocols.

### 2.7 Initial cell adhesion assay

Wells of a 24-well Primaria culture plate (Corning) were coated with iMatrix-221 (2 μL of iMatrix-221 dissolved in 420 μL PBS, 2.38 μg/mL). For preparing collagen-coated dishes, 180 mL of Milli-Q water was adjusted to pH3 using 6 N HCL, and type I collagen (FUJIFILM Wako Pure Chemical Corporation) was dissolved in this Milli-Q water to reach a final concentration of 0.3 mg/mL. Further, 5 mL of this aliquot was placed in 100-mm polystyrene culture dishes and placed in a clean bench overnight. The next day, the solution was removed and dried in a clean bench overnight [17]. Since the iMatrix-211-coated surface did not proliferate myoblasts well enough to obtain sufficient cell numbers, MACS-isolated mouse myoblasts were suspended in GM and cultured for 5 days on type I collagen-coated dishes for cell expansion. Further, cells harvested from these dishes by trypsinization were seeded into the iMatrix-221-coated 24-wells at an initial cell density of 7300 cells per well and cultured in GM at 37 °C in a humidified CO_2_ incubator. At the denoted time points, the culture medium containing floating, non-adhered cells was removed. Cells adherent to the wells were gently washed with PBS and subjected to automated cell counting using a Confocal High Content Screening System, Image Xpress Ultra (Molecular Devices) after cell nuclei were stained with DNA-binding fluorescent dye (Hoechst 33258). Cell counting was performed only in the central portion (5.6 mm^2^) of the wells, which could be specified by the software. Three independent experiments from three different mice were performed.

### 2.8 Primary mouse myoblast isolation with iMatrix-221-coated culture surfaces

A total of 1 g collected mouse muscle was obtained by the aforementioned method. First, 50 mg of collagenase type II (Worthington Biochemical Corporation, Lakewood, USA) was dissolved in 100 mL HBSS(−) and 20 mL each was transferred into five 50 mL tubes. As an inhibitor to stop the reaction with collagenase, four 50 mL tubes containing 20 mL of HBSS(−) were prepared and kept in ice-cold conditions. The collected muscles were minced, placed in the collagenase solution, and incubated in a 37 °C thermostatic bath for 10 min without shaking. The supernatant was discarded, and 20 mL of the prepared collagenase solution was re-added and incubated for 10 min with shaking at 130–80 rpm. A 40-μm strainer was set on a 50 mL tube containing HBSS(−) stored in ice, supernatant was filtered, and the tube was stored in ice again. The next 20 mL of collagenase solution was placed in the tube with the remaining minced muscle tissue and incubated at 37 °C for 10 min with shaking. This process was repeated three more times. The last filter may easily be clogged because the last suspension contains remaining muscle tissue; thus, the tissue was gently pressed by a cell scraper. We obtained four 50 mL tubes containing the collected suspension, which were then centrifuged at 4 °C and 300×*g*, for 10 min. The obtained cell pellet was resuspended with 10 mL GM without FBS, and all cells were seeded in one iMatrix-221 or non-coated 100-mm Primaria cell culture dishes. After culture at 37 °C for 2 h in a humidified CO_2_ incubator, floating non-adhered cells were removed by medium change. Cells adhered on the iMatrix-221-coated and non-coated surfaces were subjected to prolonged culture on the same surfaces for about one week. Then, cells were subjected to immunofluorescence staining after labelling with anti-desmin antibody as well as a DNA-binding dye, and the desmin-positive cells were counted.

### 2.9 Cell proliferation assay of primary mouse MACS-isolated myoblasts on various materials-coated dishes

Wells of 24-well Primaria culture plates were coated with either iMatrix-221 (2 μL of iMatrix-221 dissolved in 420 μL PBS, 2.38 μg/mL), type I collagen (420 μL of aliquot prepared in the initial cell adhesion assay, 0.3 mg/mL), or Matrigel® (Corning, 10 μL of Matrigel dissolved in 200 μL PBS, 450 μg/mL) [18]. After these aliquots were added, they were incubated for 1 h at 37 °C in a humidified 5% CO_2_ incubator. Control wells were without any coating. MACS-isolated mouse skeletal myoblasts were centrifuged, and pellets were resuspended in 3.6 mL of GM. Aliquots (100 μL) of this cell suspension were seeded into the wells and cultured in GM at 37 °C in a humidified 5% CO_2_ incubator. At the denoted time points, the cells in the wells were subjected to automatic cell counting using a Confocal High Content Screening System, Image Xpress Ultra (Molecular Devices), after cell nuclei were stained with a DNA-binding fluorescent dye (Hoechst 33258). Culture wells were 15.49 mm in diameter, and the central portion (5.6 mm^2^) was counted since the well's bottom was cup-shaped and cells easily gathered into the center. This experiment was performed independently three times (from three separate mice), and each experiment was performed in triplicate. Each denoted symbol on Fig. 5 shows the average and SEM of cell numbers on the total of nine wells. The maximum three values of the number of cells obtained on each culture surface, regardless of the time from the primary culture, were averaged and subjected to Dunnett's test using GraphPad Prism software version 8 (GraphPad Software, San Diego, Canada).

### 2.10 Immunofluorescence staining

MACS-isolated mouse primary cultured myoblasts were also cultured on type I collagen-coated dishes until they reached 80% confluence, and then, were harvested from these dishes by trypsinization. Harvested cells were then seeded onto wells of 4-well slide chambers (Nunc™ Lab-Tek™ II Chamber Slide™, Life Technologies) at an initial cell density of 60,000 cells/well. The slide chambers were incubated at 37 °C overnight to allow cell adhesion, and for counting desmin-positive cells, PFA (FUJIFILM Wako Pure Chemical Corporation) fixation was performed. Next, for the differentiation experiment, in another slide chamber with obtained cells, culture medium was changed to differentiation medium the next day, and the cells were cultured for 7 days and PFA-fixed. Mouse thigh skeletal muscle was frozen in isopentane at the temperature of liquid nitrogen. Then, 8-μm-thick cryo-tissue sections were prepared using a cryostat, fixed with acetone, and subjected to immunofluorescence staining. Cultured cells were fixed with 4% PFA in PBS for 15 min. After washing with PBS, the cells and tissue sections were both incubated with 0.5% Triton-X (Sigma-Aldrich) in PBS for 10 min for permeabilization, washed with PBS, blocked using Blocking One Histo (NACALAI TESQUE, Tokyo, Japan) for 15 min, incubated with primary antibodies (rabbit anti-desmin or rabbit anti-fast myosin skeletal heavy chain antibodies, 1:100 diluted, Abcam, Cambridge, UK), washed, and incubated with Alexa-Fluor 488-conjugated anti-rabbit IgG antibody (1:200 diluted, Abcam). The immunostained cells and tissue sections were observed using a confocal laser scanning fluorescence microscope (FV1200, Olympus, Tokyo, Japan) and Cell Sens Standard software (FV1-ASW, Olympus, Tokyo, Japan). Cell nuclei in the desmin-positive cells were manually counted. Myoblast purity was obtained by dividing the number of desmin-positive cells by the total cell number in randomly selected five fields of view. Errors were expressed as ± SEM with n = 5 in mouse and n = 3 in rat.

### 2.11 Gene expression analyses of primary myoblasts

Mouse primary myoblasts were isolated by either of two methods (i.e. MACS and iMatrix-221-method). In the iMatrix-221-method, the total cell population from enzymatically digested mouse skeletal muscle was plated on iMatrix-221-coated dishes and allowed to adhere in GM for 2 h at 37 °C. Then, the non-adherent cells were removed. Adherent cells were subjected to prolonged culture on the same surfaces. Primary cells adherent on iMatrix-221-coated dishes and MACS-isolated myoblasts were cultured on both iMatrix-221-coated dishes and type I collagen-coated dishes, respectively, until they reached 80% confluence (about one week). Cell cultures were then subjected to total RNA isolation and gene expression analyses by TaqMan PCR. Total RNA was purified with RNeasy plus mini kit (QIAGEN, Venlo, Netherlands), according to the manufacturer's protocol. Further, cDNA was synthesized and RT-qPCR was performed using TaqMan Fast Advanced Master Mix and TaqMan probes for *Pax 7*, *MyoD*, *GATA4*, *GAPDH*, and *myf5*. These specific probes were provided by Life Technologies. mRNA expression was evaluated by comparing the expression level of each mRNA to that of *GAPDH*.

### 2.12 *Integrin α7X2* gene expression analysis

Primary mouse skeletal muscle-derived cells adherent on iMatrix-221-coated dishes and not-adherent floating cells were separated after 2-h incubation at 37 °C. Non-adherent floating cells were transferred onto type I collagen-coated dishes. Both cells were cultured in GM until they reached 80% confluence. Expanded cells were harvested from these surfaces by trypsinization and subjected to gene expression analysis for *integrin α7X2* and *GAPDH* genes with the specific TaqMan probes. Results of RT-qPCR were evaluated by the ΔΔCt method.

### 2.13 Primary rat myoblast isolation with iMatrix-221-coated dishes

Rat skeletal muscle-derived cell populations were prepared as described above. Then, the total cells were seeded onto 100-mm Primaria dishes coated with iMatrix-221 and cultured in GM without FBS at 37 °C for 2 h in a humidified CO_2_ incubator. As a negative control, dishes without iMatrix-221 coating were also used. Then, floating non-adherent cells were removed by medium change. The remaining cells adhered on the iMatrix-221-coated surfaces were subjected to prolonged culture on the same surfaces. After reaching 80% confluence, both cell cultures were harvested, re-seeded in slide chambers, and subjected to immunofluorescence staining with anti-desmin antibody and a DNA-binding dye for cell nuclei staining using the same protocol as that used for mouse samples.

## 3 Results

### 3.1 Initial cell adhesion of mouse MACS-isolated myoblasts on iMatrix-221-coated surfaces

**Fig. 1:**
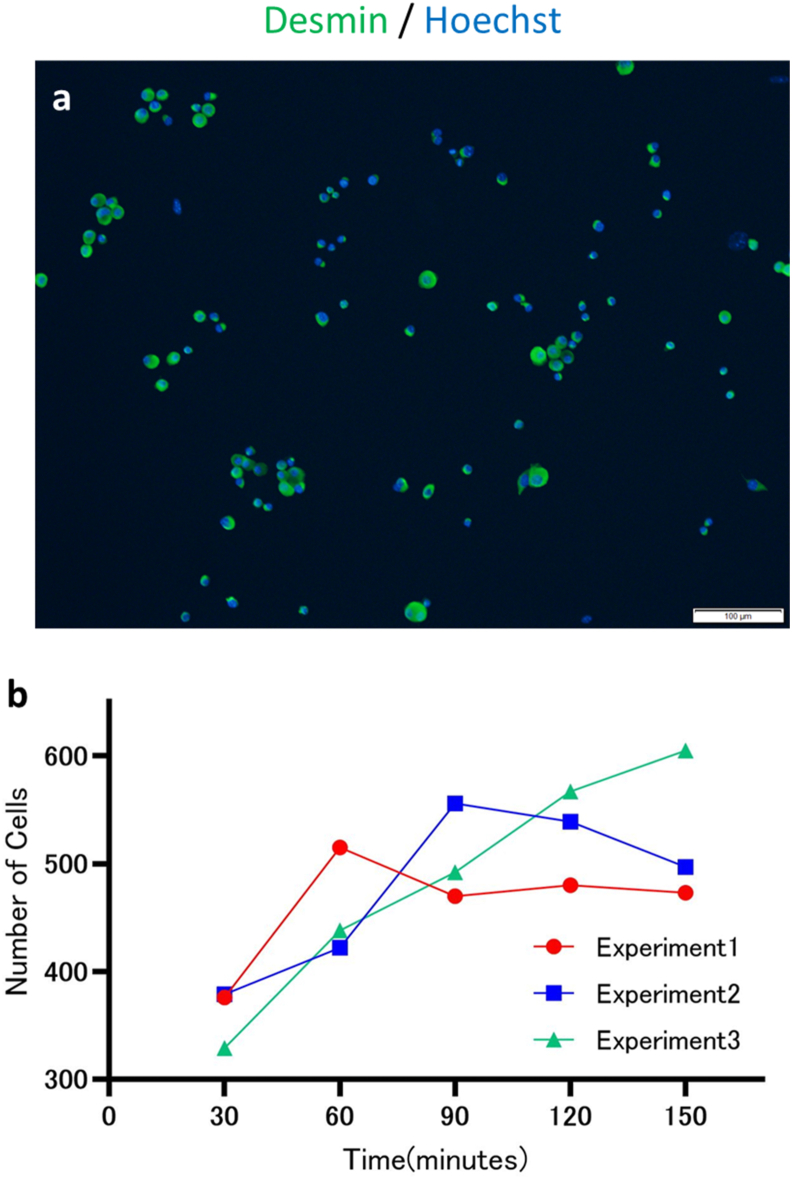
MACS-isolated primary mouse skeletal myoblasts were expanded on type I collagen-coated dish, and seeded into 24-well plates coated with iMatrix-221 (7300 cells per well) and incubated in a humidified 5% CO_2_ incubator at 37 °C. At the denoted time points, non-adherent, floating cells were removed by medium change and washing with PBS. The remaining adherent cells were subjected to nuclei staining with a DNA-fluorescent dye, and the number of adherent cells was automatically counted. (a) Immunofluorescence staining of MACS-isolated myoblasts with anti-desmin antibody revealed that the desmin-positive cell rate of MACS-isolated cells used in the present study was 98.5% ± 0.208 (n = 3). (b) The adhesion of primary myoblasts to iMatrix-221-coated surfaces was observed even from 30 min after cell seeding. Scale bar = 100 μm.

MACS-isolated primary myoblasts were expanded on type I collagen-coated (27 μg/cm^2^) dishes. They were examined by immunofluorescence staining with anti-desmin antibody to confirm myoblast purity after expansion. Desmin-positive and total cell numbers were manually counted, and the ratio of desmin-positive cells to total cells was 98.5 ± 0.208% (n = 3) (Fig. 1a). These cells were seeded on iMatrix-221-coated 24 well Primaria plate. Initial cell adhesion was evaluated by counting the adhered cells in a time-dependent manner (Fig. 1b). After culture until the denoted time points, floating, non-adherent cells were removed, and adherent cells were fixed and stained with a DNA-binding dye. Then, cell nuclei were automatically counted. Almost all MACS-isolated myoblasts exhibited a spherical morphology on iMatrix-221-coated surfaces under phase contrast microscopy, but even after gentle washing with PBS, a portion of the cells remained on the surfaces. Automated cell counting after cell nuclei staining revealed that even at early time points (30 min), a portion of the seeded cells stably adhered on the surfaces and increased over time after cell seeding (Fig. 1b).

### 3.2 Primary culture of mouse myoblasts isolated on iMatrix-221-coated dishes and muscle differentiation

**Fig. 2:**
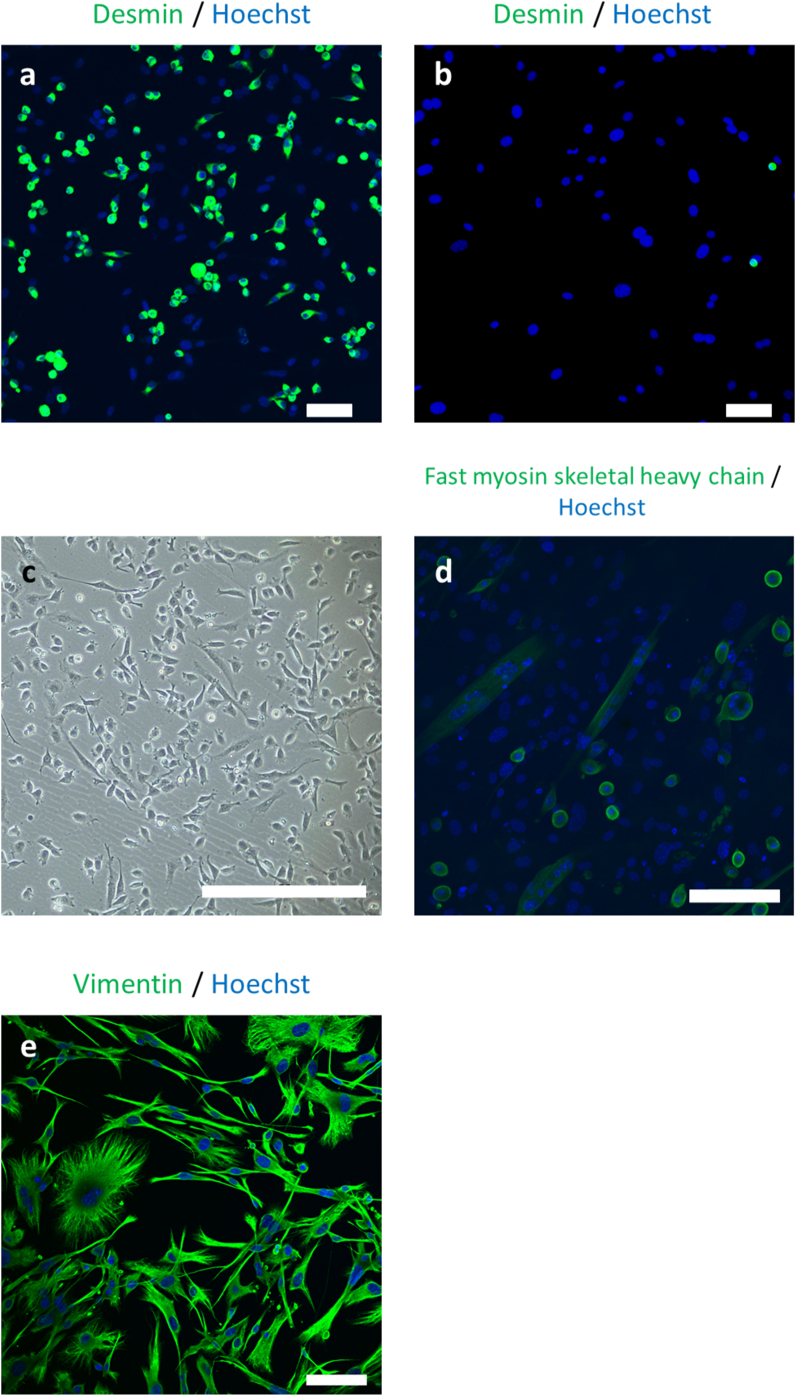
Primary culture of mouse myoblasts isolated on the iMatrix-221-coated dishes. The total cell populations from enzymatically digested minced fresh mouse skeletal muscle were directly seeded on iMatrix-221-coated dishes. After 2-hour culture at 37 °C, the non-adherent cells were removed, and the adherent cells were subjected to prolonged culture on the same surface until they reached 80% confluence. Cells harvested by trypsinization were re-seeded in slide chambers and observed after immunofluorescence staining. (a) Immunofluorescence staining of mouse cells obtained with iMatrix-221-coated dishes: 70.3 ± 5.49% (n = 5) of cells were desmin positive. In contrast, (b) cells adherent on control dishes without iMatrix-221 coating were 7.24% desmin-positive, implying these cells were mostly fibroblasts. (c) Phase contrast microscopic images of cells after expansion. (d) Immunofluorescence staining showed that some cells formed multinucleated myotubes positive for fast myosin skeletal heavy chain after incubation with DM. (e) Not adherent cells were cultured on collagen dish. Immunofluorescence staining of these cells revealed that they were all vimentin-positive cells. Scale bars = 100 μm.

Fig. 2a shows that 70.3 ± 5.49% (n = 5) cells isolated with iMatrix-221-coated dishes from murine primary muscle tissues and cultured directly on iMatrix-221-coated dishes were desmin-positive. In contrast, cells adhered on control dishes without iMatrix-221-coating were 7.24% desmin-positive (Fig. 2b), implying that these cells are mostly fibroblasts. The obtained cells showed a spindle shape under a phase contrast microscope (Fig. 2c). In addition, after culture of cells adhered on iMatrix-221-coated surfaces, cells were further cultured in GM on slide chambers for a day. Seven days after changing the medium to DM, spindle-shaped multinuclear cells showing spontaneous cell contraction were observed under a phase contrast microscope (Supplementary video file-mouse_myotube.mp4). Immunofluorescence staining with anti-myosin skeletal heavy chain-antibody revealed that these cells were fast myosin skeletal heavy chain-positive (Fig. 2d), suggesting that myoblasts that had a potential to differentiate to mature muscle cells were successfully isolated on iMatrix-221-coated surfaces even without MACS.

Supplementary video related to this article can be found at https://doi.org/10.1016/j.reth.2022.04.006

The following is the supplementary data related to this article:

Video 1 Video 1

### 3.3 Expression of skeletal myogenesis-related genes by primary mouse myoblasts isolated using iMatrix-221-coated dishes

**Fig. 3:**
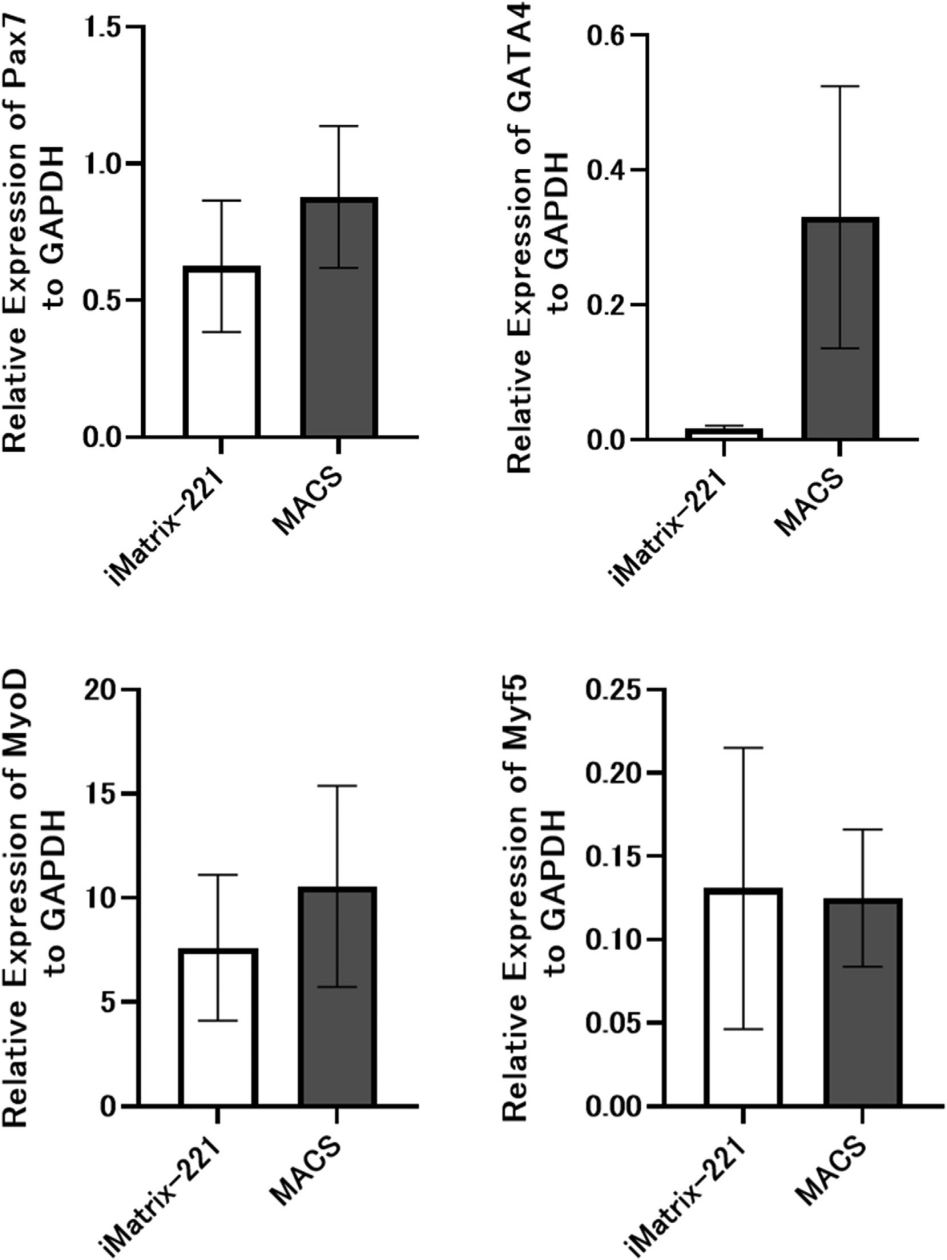
RT-qPCR analysis of cell expression of myogenesis-related genes *Pax 7*, *MyoD*, *GATA4*) and *Myf5* compared with *GAPDH* in isolated primary skeletal myoblasts. Myoblasts obtained with the MACS method were analyzed as control. Both cells showed a certain level of *Pax7* expression, indicating that they exhibited characteristics of myoblasts, although their expression levels are different in *GATA4* and *MyoD*. Bars represents the mean, and the thin lines represent SEM of the three independent experiments (n = 3).

Skeletal myogenesis-related gene expression by primary mouse myoblasts isolated on iMatrix-221-coated surfaces was quantitatively evaluated by TaqMan PCR. Four skeletal myogenesis-related genes were examined, and the results with primary myoblasts isolated by MACS are also shown (Fig. 3). *Pax 7*, a transcriptional factor gene that plays a role in myogenesis, commonly used as a marker for muscle satellite cells showed a similar relative expression level in primary myoblasts isolated by the iMatrix-221-method to that of MACS-isolated primary myoblasts, indicating the presence of satellite cells in the cell population. Expression levels of *GATA4*, a transcriptional factor responsible for myocardial differentiation and function, and *MyoD*, also a transcriptional factor that represses satellite cell renewal and promotes terminal differentiation, were detected in primary myoblasts isolated by iMatrix-221-method. All transcription factor expression showed no significant differences by paired t-test, but GATA4 exhibited a tendency to be low in myoblasts isolated by iMatrix-221-method. Similar levels of expression of *myf5*, which regulates skeletal muscle differentiation and myogenesis, were observed in both myoblast cultures.

### 3.4 Expression of integrin α7X2 gene by primary mouse myoblasts isolated using iMatrix-221-coated dishes

**Fig. 4:**
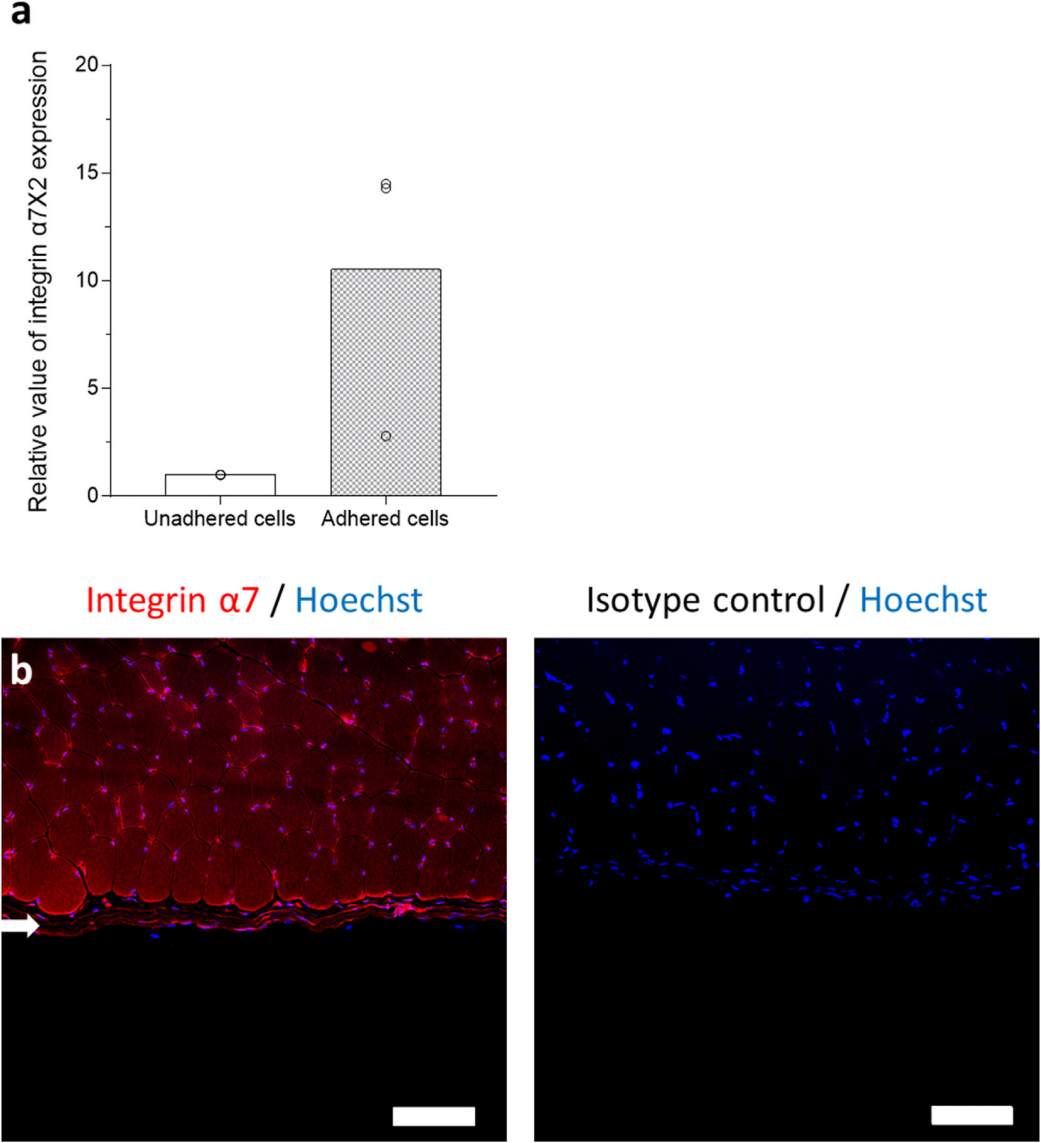
RT-qPCR analysis of mRNA expression of *integrin α7X2* in adherent primary cells on iMatrix-221-coated dishes versus non-adherent floating cells cultured on type I collagen-coated dishes. (a) Bar graph of the relative value of *integrin α7X2* expression. Adherent cells seeded in the iMatrix-221-coated dishes and not-adherent cells seeded in the type I collagen-coated dishes were incubated until they reached 80% confluence and were evaluated by RT-qPCR (ΔΔCt method with *GAPDH* house-keeping gene). Adherent cells showed 10.5-fold higher *integrin α7X2* expression compared to not-adherent cells (n = 3). Bars represent the mean, and the dots represent the relative value. (b) Immunohistochemical staining of mouse tibialis anterior muscle with anti-integrin α7 antibody. The periphery of the muscle fibers was positively stained, but some faintly stained cells in the fascia (arrow) are also evident. (c) Neither myocytes nor fibroblasts showed positive staining when isotype control antibody was used as the primary antibody. Scale bars = 100 μm.

To confirm that cell binding onto iMatrix-221-coated surfaces is mediated by a specific integrin, cell mRNA expression of the *integrin α7X2* gene was quantitatively evaluated in adherent and not-adherent floating cells. Although no significant difference was found between expression levels of these cells by paired t-test, expression was 10.5 times higher in cells adhered on iMatrix-221-coated surfaces than in not-adherent cells (Fig. 4a). Immunofluorescence staining of skeletal muscle tissue with anti-α7 integrin antibody revealed that the periphery of each myotube was positively stained, and some positively stained cells were also observed in adjacent fascia (Fig. 4b and Supplementary Fig. 1), implying that fibroblasts in fascia also expressed this integrin in relatively low amounts. In contrast, neither myocytes nor fibroblasts showed positive staining when isotype control antibody was used as the primary antibody (Fig. 4c).

### 3.5 Cell proliferation assay of primary MACS-isolated mouse myoblasts on various coated culture surfaces

**Fig. 5:**
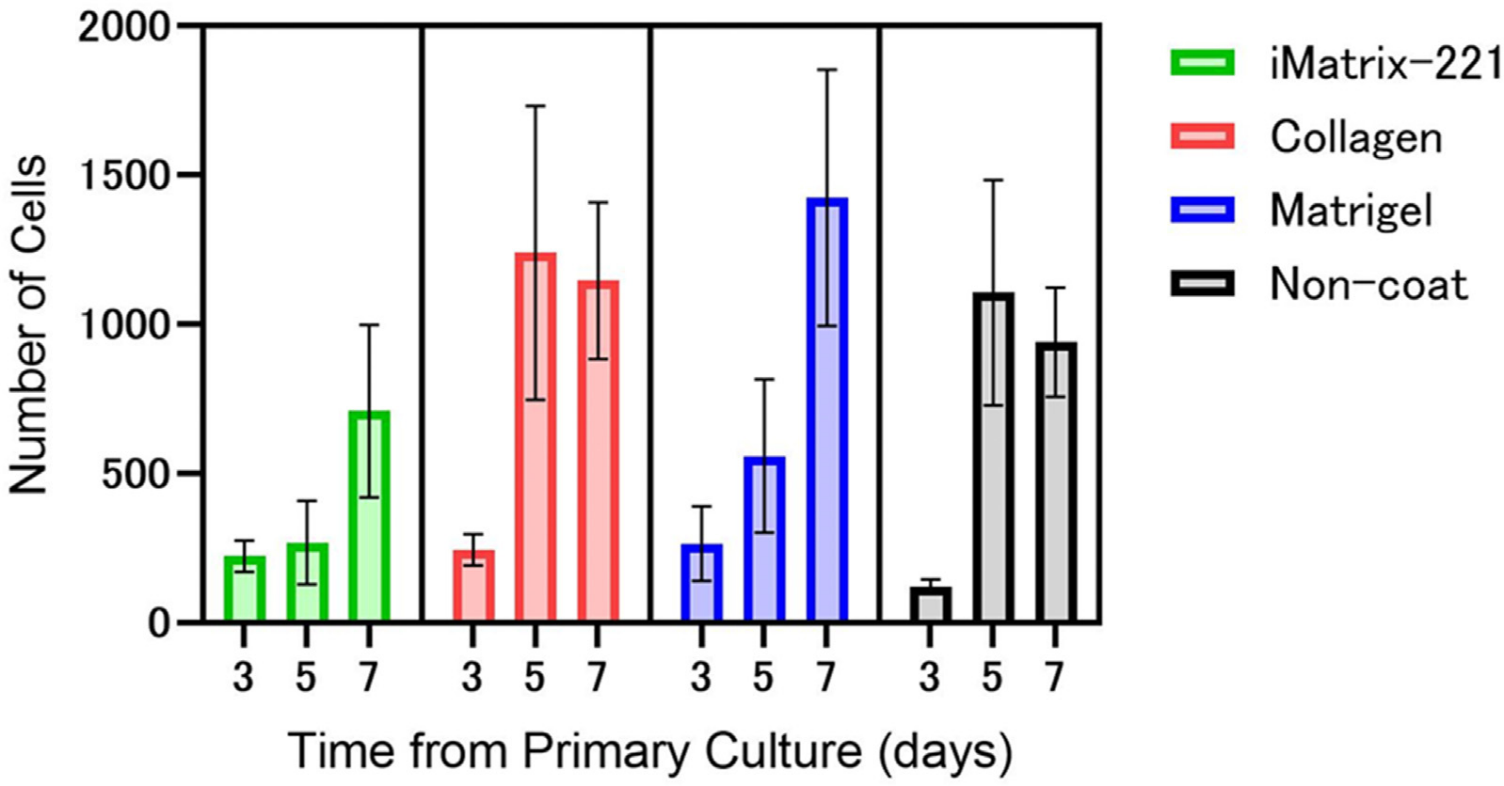
Proliferation of MACS-isolated primary mouse skeletal myoblasts on culture surfaces coated with iMatrix-221, type I collagen, or Matrigel®. Primary mouse skeletal myoblasts isolated by MACS and expanded on type I collagen-coated dishes were seeded onto three different wells at same seeding density and counted on Day 3, 5, and 7 in GM. Uncoated wells served as controls. Three independent experiments from three mice were performed (average and SEM of nine culture wells per treatment shown). The bold line represents the mean, and the thin lines represent SEM. The number of cells increased the most in the type I collagen-coated dishes and the least in the iMatrix-221-coated dishes; however, no significant differences in cell count were observed among these coated materials.

MACS-isolated primary mouse myoblasts were cultured on iMatrix-221-coated surfaces, and the proliferation was compared with those on various material-coated surfaces (Fig. 5). Cell numbers increased over time. The number of cells increased the most in the collagen-coated dish and the least in the iMatrix-221-coated surface, but statistical analysis revealed that no significant differences were observed among coated culture materials.

### 3.6 Cells obtained from rat skeletal muscle with iMatrix-221-coated dishes

**Fig. 6:**
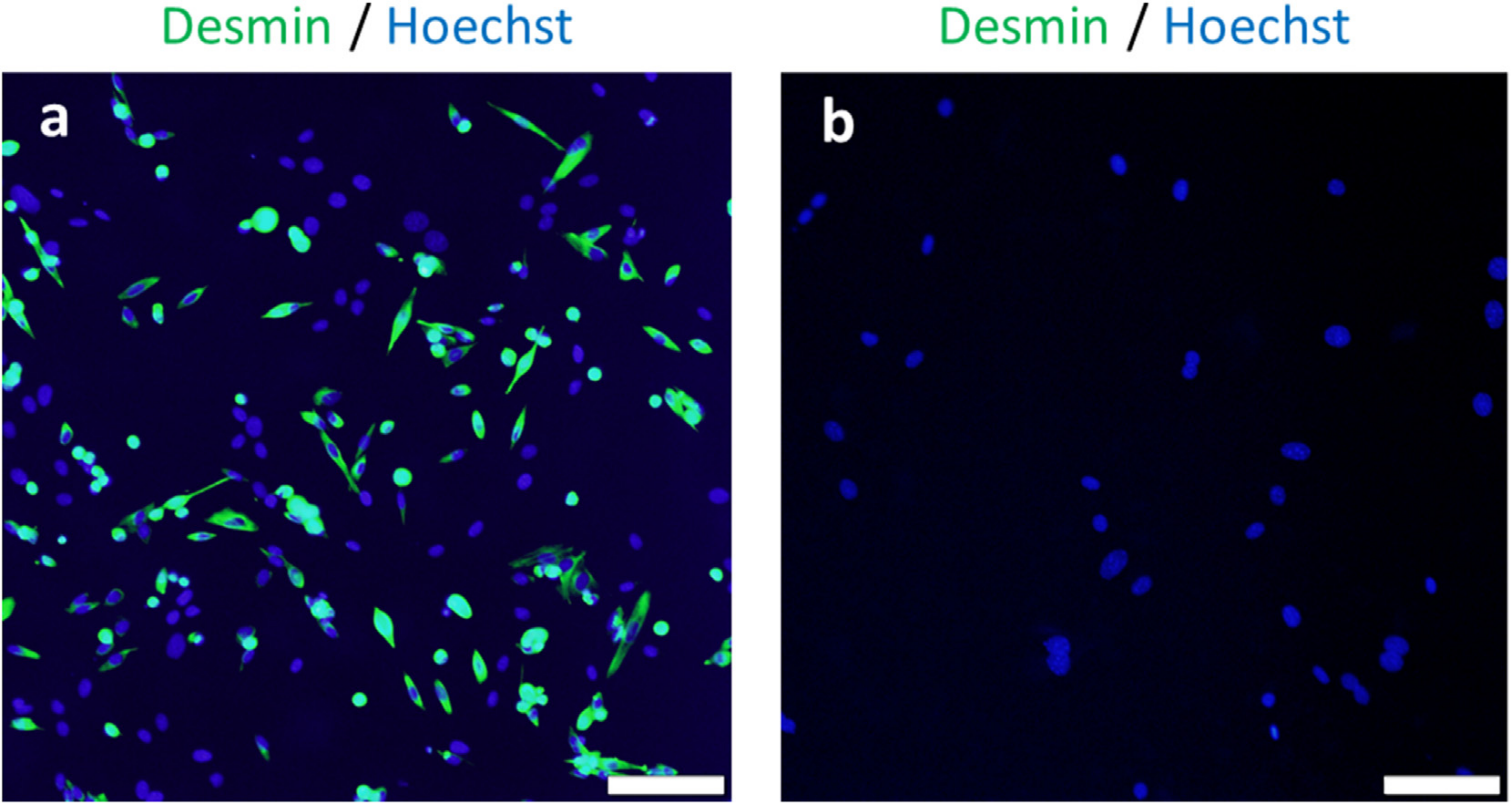
Primary culture of rat myoblasts on iMatrix-221-coated dishes. Cells obtained from rat skeletal tissue isolates and cultured on Imatrix-221-coated dishes. (a) Numbers of desmin-positive cells obtained were 67.7 ± 1.65% (n = 3) and (b) those in the uncoated culture control were 0.613%. Scale bars = 100 μm.

Finally, to show that the present method to isolate skeletal myoblast with the laminin-221 fragment is species-independent, myoblast populations obtained from rat skeletal muscle were plated on either iMatrix-221-coated dishes or non-coated Primaria dishes, and cultured for 2 h in GM. Then, the non-adhered floating cells were removed by media changes and gentle washing with PBS. Adhered cells were subjected to prolonged culture on the same surfaces. Immunofluorescence staining with anti-desmin antibody revealed that 67.7 ± 1.65% (n = 3) of cells isolated using the iMatrix-221 method were desmin-positive skeletal myoblasts (Fig. 6a). In contrast, cells adhered on non-coated dishes were 0.613% desmin-positive, implying that these cells were mostly fibroblasts adhered to surfaces without iMatrix-211 coating via endogenous fibronectin contained in GM (Fig. 6b).

## 4 Discussion

The aim of the present study was to establish a facile method for primary culture of myoblasts. Previously, primary myoblast isolation has been performed using several methods such as tissue-explant, separation using Percoll density gradients, and antibody-based FACS and MACS [[12], [13], [14],^19^]. A primary culture method using differences in cell adhesion capacity has also been reported [16]. The method utilized the rapid adhesion of unwanted f`ibroblasts to collagen-coated dish to eliminate these cells; the supernatant containing non-adhered, floating myoblasts was collected 2 h after seeding, and subjected to prolonged culture. In the present study, we assumed that myoblasts would adhere to laminin-221 fragment-coated dishes more rapidly than fibroblasts, and that the albumin-coating of bare plastic dish surfaces would hinder fibroblasts from adhesion onto these surfaces. Because the initial adhesion of MACS-isolated primary myoblasts adhered on iMatrix-221-coated dishes occurs within 2 h, floating cells, which were assumed to be fibroblasts, were removed after 2-hour incubation, and the adherent cells were subjected to prolonged culture on the same surfaces. The initial 2-hour incubation needs growth medium lacking the cell adhesion protein, fibronectin, to produce only laminin-dependent cell adhesion. With this protocol, 70.3% desmin-positive cells were obtained, and immunofluorescence staining confirmed that these cells were both multinucleated and fast myosin skeletal heavy chain-positive after culture in DM (Fig. 2d). Desmin-positive cells and their differentiative potency suggested that the cells isolated by the present method were myogenic progenitors [20].

RT-qPCR comparisons between cells adhered on iMatrix-221-coated dishes and non-adhered cells revealed that the expression level of *integrin α7X2* was 10.5-fold higher in adherent cells, suggesting that cell adhesion to the surfaces were integrin-mediated. Laminin-derived recombinant fragments have been utilized as culture substrates for various cell types including human induced pluripotent stem cells and embryonic stem cells [7]. Besides, they have been utilized for differentiation of various cell types such as keratinocytes [21], neuronal cells [22], cardiac [7] and skeletal myocytes [23], and ocular cells [24]. However, this is the first report to utilize these recombinant fragments for cell isolation. Non-adherent cells exposed to iMatrix-221-coated surfaces were vimentin-positive (Fig. 2e), implying these were fibroblasts. The observed expression of integrin α7 in a small number of cells in fascia (Fig. 4b) might explain why the purity of desmin-positive cells isolated on iMatrix-221-coated dishes in this study did not reach 100%.

RT-qPCR of the obtained cells showed expression of *Pax 7*, a transcription factor of satellite cells [25], as well as in myoblasts sorted by the MACS method used as controls. In addition, *MyoD* and *GATA4* [26,27], the transcription factors expressed during skeletal muscle differentiation, were also found to be expressed in both iMatrix-221 cultured cells and MACS control cells. In these transcription factors, GATA4 expression was especially low in iMatrix-221 cultured cells. GATA4 is known as a transcription factor that delays muscle differentiation [28], therefore, low GATA4 expression may indicate that differentiation was accelerated. This is consistent with the experimental results that cell proliferation on iMatrix-221-coated culture dishes was slower than on other coatings. *Myf5* is a transcription factor involved in muscle regeneration [29] and is reported to promote higher transplantation efficiency when it is highly expressed [30]. Cultured cells isolated by the present method produced equal or higher phenotypic marker expressions compared to cells obtained by MACS. These findings lead to the conclusion that iMatrix-221 cultured cells can be used in further studies of myoblasts, myogenesis, and for myoblast transplantation in basic studies as well as possible clinical applications.

Notably, the new iMatrix-221-method would leverage the use of primary myoblasts in cell therapy, tissue engineering, and regenerative medicine by reducing the time and cost required for cell preparation in culture because this method enables continuous workflows from cell isolation, proliferation, and differentiation. Recombinant fragments of the laminin E8 domain (*e.g.* iMatrix-221) produced by transfected Chinese Hamster ovary cells are less expensive and product purity is reasonably high [31]. Therefore, for cell isolation, culture, and differentiation, these recombinant fragments are attractive for use as a culture substrate. Myoblasts cultured on iMatrix-221-coated surfaces as a regenerative source for muscle diseases can be considered for clinical applications in future. For further clinical application, E8 fragments should be immobilized onto cell culture surfaces, not absorbed from media.

Although a commercial MACS kit for mice did not work with rat myoblasts (failure in this isolation might be due to the species specificity of the antibody agents used), the present iMatrix-221 culture method seemed to have no limitations in terms of its utility for the two animal species.

## 5 Conclusion

We established a new facile method for primary culture of myoblasts from mouse and rat skeletal muscles by exploiting the high affinity of integrin α7X2β1 to laminin-221. The number of desmin-positive cells in this method did not reach 100%, possibly because of the expression of *integrin α7X2* in fibroblasts derived from the fascia. The binding of integrin to laminin is known to promote cell differentiation and proliferation, but for laminin-221, it did not promote myoblast proliferation.

## Author contributions

Y.K. designed the study, performed the experiments, analyzed the data, and drafted the manuscript. J.H. and R.T. advised on experimental method. M.Y., N.S and K.I. conceived of the study and supervised all experiments. All authors critically revised the report, commented on the manuscript, and approved the final submission content.

## Declaration of competing interest

iMatrix-221 was provided by Nippi. Inc., Tokyo, Japan. MY received Consulting honorarium from Nippi. Inc.

## Figures and Tables

**figs1:**